# Role of sodium‐dependent Pi transporter/Npt2c on Pi homeostasis in klotho knockout mice different properties between juvenile and adult stages

**DOI:** 10.14814/phy2.14324

**Published:** 2020-02-05

**Authors:** Ai Hanazaki, Kayo Ikuta, Shohei Sasaki, Sumire Sasaki, Megumi Koike, Kazuya Tanifuji, Yuki Arima, Ichiro Kaneko, Yuji Shiozaki, Sawako Tatsumi, Tomoka Hasegawa, Norio Amizuka, Ken‐ichi Miyamoto, Hiroko Segawa

**Affiliations:** ^1^ Department of Applied Nutrition Institute of Biomedical Sciences Tokushima University Graduate School Tokushima Tokushima Japan; ^2^ Developmental Biology of Hard Tissue Hokkaido University Graduate School of Dental Medicine Sapporo Japan

**Keywords:** FGF23/klotho, growth, intestine, kidney, phosphate transporter

## Abstract

SLC34A3/NPT2c/NaPi‐2c/Npt2c is a growth‐related NaPi cotransporter that mediates the uptake of renal sodium‐dependent phosphate (Pi). Mutation of human NPT2c causes hereditary hypophosphatemic rickets with hypercalciuria. Mice with Npt2c knockout, however, exhibit normal Pi metabolism. To investigate the role of Npt2c in Pi homeostasis, we generated α‐klotho^−/−^/Npt2c^−/−^ (KL2cDKO) mice and analyzed Pi homeostasis. α‐Klotho^−/−^ (KLKO) mice exhibit hyperphosphatemia and markedly increased kidney Npt2c protein levels. Genetic disruption of Npt2c extended the lifespan of KLKO mice similar to that of α‐Klotho^−/−^/Npt2a^−/−^ mice. Adult KL2cDKO mice had hyperphosphatemia, but analysis of Pi metabolism revealed significantly decreased intestinal and renal Pi (re)absorption compared with KLKO mice. The 1,25‐dihydroxy vitamin D3 concentration was not reduced in KL2cDKO mice compared with that in KLKO mice. The KL2cDKO mice had less severe soft tissue and vascular calcification compared with KLKO mice. Juvenile KL2cDKO mice had significantly reduced plasma Pi levels, but Pi metabolism was not changed. In Npt2cKO mice, plasma Pi levels began to decrease around the age of 15 days and significant hypophosphatemia developed within 21 days. The findings of the present study suggest that Npt2c contributes to regulating plasma Pi levels in the juvenile stage and affects Pi retention in the soft and vascular tissues in KLKO mice.

## Graphical abstract

SLC34A3/NPT2c/NaPi‐2c/Npt2c is a growth‐related NaPi cotransporter that mediates the uptake of renal sodium‐dependent phosphate (Pi). Mutation of human NPT2c causes hereditary hypophosphatemic rickets with hypercalciuria. Npt2c contributes to regulating plasma Pi levels in the juvenile stage and affects Pi retention in the soft and vascular tissues in KLKO mice.

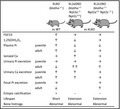

## 1 INTRODUCTION

The serum inorganic phosphate (Pi) concentration is controlled by the intestines and kidneys, which regulate Pi absorption/excretion; the bones, liver, and muscles, which store Pi; and regulatory factors (Kaneko, Tatsumi, Segawa, & Miyamoto, 2017). Sodium‐dependent phosphate transporters (the solute carrier 34 [SLC34] family) have an important role in regulating serum Pi levels. SLC34A1/NPT2A/NaPi‐2a and SLC34A3/NPT2C/NaPi‐2c are predominantly expressed in the proximal tubules of the kidney (Kaneko et al., 2011; Lederer & Miyamoto, 2012; Levi et al., 2019). The phenotypic differences resulting from NPT2A and NPT2C gene mutations may be due to differential dominance of the transporters involved in Pi homeostasis. In humans, NPT2C mutations cause hereditary hypophosphatemic rickets with hypercalciuria (HHRH), a primary renal Pi wasting disorder that results in increased serum 1,25‐dihydroxy vitamin D3 [1,25(OH)_2_D_3_] concentrations with associated intestinal Ca^2+^ hyperabsorption, hypercalciuria, and rickets/osteomalacia (Bergwitz & Miyamoto, 2019; Bergwitz et al., 2006; Lorenz‐Depiereux et al., 2006; Yamamoto et al., 2007). In mice, disruption of Npt2c (Npt2 designate a mouse NaPi‐2) causes hypercalciuria and increased serum 1,25(OH)_2_D_3_ concentrations, but not hypophosphatemia, rickets, or nephrocalcinosis (Segawa, Onitsuka, Kuwahata, et al., 2009). Furthermore, only Npt2a/Npt2c double knockout (KO) mice exhibit a physiology similar to that of patients with HHRH (Segawa, Onitsuka, Furutani, et al., 2009). In rodents, Npt2a is a major functional NaPi cotransporter in the proximal tubules (Kaneko et al., 2017; Lederer & Miyamoto, 2012; Levi et al., 2019). Npt2c is important for Pi reabsorption in weanling animals but mediates a very small percentage of Pi reabsorption in adult animals (Segawa et al., 2002). A recent study demonstrated that mice with kidney‐specific Npt2c deletion show no prominent phenotype (Myakala et al., 2014). The Npt2c transcript, however, was detected in several tissues, suggesting that extrarenal Npt2c has an important role (Kuwahara et al., 2012; Nishimura & Naito, 2008).

Fibroblast growth factor (FGF)23 is a hormone that promotes renal Pi excretion by decreasing Pi reabsorption in the proximal tubules while concurrently reducing the plasma 1,25(OH)_2_D_3_ concentration by both decreasing its biosynthesis and increasing its metabolism (Hu, Shi, & Moe, 2019). FGF23 requires an additional cofactor, α‐klotho, to bind with high affinity and signal efficiently through its cognate FGF receptors. Klotho and FGF receptors form a heterodimeric receptor for FGF23 (Urakawa et al., 2006). α‐Klotho‐mutant (kl/kl) mice exhibit blunted FGF23 signaling and increased Pi reabsorption, hyperphosphatemia, and ectopic calcification (Kuro‐o et al., 1997; Segawa et al., 2007). Feeding kl/kl mice a low Pi diet can rescue the kl/kl mouse phenotype because renal klotho protein is upregulated in klotho‐deficient animals (Morishita et al., 2001; Segawa et al., 2007). These findings suggest that kl/kl mice are not suitable for examining the effects of dietary Pi.

α‐Klotho knockout (KLKO) mice have a phenotype similar to that of kl/kl mice (Alexander et al., 2009; Hu et al., 2019, 2011; Kuro‐o et al., 1997; Morishita et al., 2001; Ohnishi, Nakatani, Lanske, & Razzaque, 2009a, 2009b; Segawa et al., 2007; Tsujikawa, Kurotaki, Fujimori, Fukuda, & Nabeshima, 2003; Yoshida, Fujimori, & Nabeshima, 2002). A detailed analysis of Pi metabolism in KLKO mice has not yet been reported. Ohnishi et al reported that serum Pi levels are an important determinant of calcification in α‐klotho^−/−^/NaPi2a^−/−^ mice, and lowering serum Pi levels can reduce or eliminate soft‐tissue and vascular calcification, even in the presence of extremely high serum calcium (Ca) and active vitamin D levels (Ohnishi, Nakatani, Lanske, & Razzaque, 2009a).

In the present study, we investigated the role of Npt2c in KLKO mice. Our findings revealed that although Npt2c does not affect the plasma Pi concentration, it has the same effects as Npt2a to suppress calcification and increase the lifespan in KLKO mice.

## 2 MATERIAL AND METHODS

### 2.1 Experimental animals

KLKO mice and Npt2aKO mice were purchased from CLEA Japan Inc. (Tokyo, Japan) and Jackson Laboratories, respectively. In the present study, we used KLKO mice produced by Tsujikawa et al. (2003). The generation of Npt2cKO mice was described previously (Segawa, Onitsuka, Kuwahata, et al., 2009). KLKO heterozygous mice and Npt2a or Npt2c heterozygous mice were crossed to generate KLKO (α‐klotho^−/−^, Npt2a^+/+^, Npt2c^+/+^), α‐klotho/Npt2a double KO (KL2aDKO; α‐klotho^−/−^, Npt2a^−/−^, Npt2c^+/+^), and α‐klotho/Npt2c double KO (KL2cDKO; α‐klotho^−/−^, Npt2a^+/+^, Npt2c^−/−^) mice. Genomic DNA was extracted from tail clippings and amplified by polymerase chain reaction (PCR) using the specific primers listed in Table 1. The mice were weaned at 3 weeks of age and were provided unlimited access to water and standard mouse chow (MF; Oriental). The mice were maintained under pathogen‐free conditions and handled in accordance with the Guidelines for Animal Experimentation of Tokushima University School of Medicine (T29‐3).

**Table 1:** Primers for genotyping

Application	Primer name	Primer sequence (5'‐3')
Klotho genotyping	Klotho 1	GATGGAGGCCACAGGATTGT
	Klotho 2	TGTCGCGGTAGACGTTGTTG
	Klotho 3	CGACGTTCAGACGTAGTGTG
Npt2a genotyping	Npt2a 1	ATGGCTACCACCTAGCTCCA
	Npt2a 2	CAAGTGGGATTGCTGGTTTT
	Npt2a 3	TGCTACTTCCATTTGTCACGTCC
Npt2c genotyping	Npt2c 1	CTCACCATACATGCAG
	Npt2c 2	CTGCATTTCTCAGACTCCGG
	Npt2c 3	CGGTATCGCCGCTCCCGATC

### 2.2 Metabolic cages to collect urine and fecal samples

The mice were individually placed in metabolic cages at 10:00 a.m. for quantitative urine and fecal collection for 24 hr with free access to food and water. Fecal samples were ashed according to a modified protocol (Ikuta et al., 2019; Ikuta et al., 2018). The fecal samples were collected and placed in beakers and allowed to dry at 110°C for no more than 24 hr. The samples were then ashed at 250°C for 3 hr and at 550°C for 24 hr in a muffle furnace. The samples were cooled, weighed, and digested in HCl with heat, and the sample volume was standardized to 5 ml.

### 2.3 Biochemical measurements

Plasma, urinary, and fecal Pi, and Ca were determined using commercial kits (Wako), respectively. Blood Ca^2+^ was analyzed using a Siemens Rapid Lab 348 Ca/pH analyzer. Concentrations of plasma FGF23, parathyroid hormone (PTH), and 1,25(OH)_2_D_3_ were determined using the intact FGF23 ELISA kit (KAINOS Laboratories), intact PTH ELISA kit (Immunotopics Inc.), and 1,25‐(OH)_2_ Vitamin D ELISA Kit (Immundiagnostik), respectively.

### 2.4 RNA extraction and cDNA synthesis

Total RNA was extracted from mouse tissues using ISOGEN (Wako) according to the manufacturer's instructions. After treatment with DNase (Invitrogen), cDNA was synthesized with or without the Moloney murine leukemia virus, reverse transcriptase (Invitrogen), and oligo(dT)12–18 primer. Specific primers for NaPi transporters, Cyp27A1, Cyp24A1, IL‐6, IL‐1β, and GAPDH were used for the PCR reactions. The PCR primer sequences are shown in Table 2. To check for genomic DNA contamination, a reverse transcriptase negative control experiment was performed (data not shown).

**Table 2:** Primers for RT‐PCR

Name	Sense primer sequences (5'‐3')	Anti sense primer sequence (5'‐3')
1aOHase(qPCR)	GAGCAAACTCCAGGAAGCAG	TGAGGAATGATCAGGAGAGG
240Hase (qPCR)	TGGGAAGATGATGGTGACCC	TCGATGCAGGGCTTGACTG
Npt2a (qPCR)	AGTCTCATTCGGATTTGGTGTCA	GCCGATGGCCTCTACCCT
Npt2c (qPCR)	TAATCTTCGCAGTTCAGGTTGCT	CAGTGGAATTGGCAGTCTCAA
Npt2c (semi‐quantitative PCR)	AGGTCCCCAACCCTACTCTG	TGCCTAGTAGCTGGAAAGCA
Npt2b (qPCR)	CCTGGGACCTGCCTGAACT	AATGCAGAGCGTCTTCCCTTT
PiT1 (qPCR)	CCCATGGACCTGAAGGAGGA	GCCACTGGAGTTGATCTGGT
PiT2 (qPCR)	CTCAGAAGGCACGTCAGCAG	AAACGTGACCGTCATTCCTC
IL‐6 (qPCR)	CACAAGTCCGGAGAGGAGAC	TTGCCATTGCACAACTCTTT
IL‐lb(qPCR)	TGCCACCTTTT GACAGTGATG	ATGTGCTGCTGCGAGATTTG
GAPDH (qPCR)	CTGCACCACCAACTGCTTAGC	CATCCACAGTCTTCTGGGTG
GAPDH (semi‐quantitative PCR)	i CTGCACCACCAACTGCTTAGC	GCCTGCTTCACCACCTTCTTG

### 2.5 Protein sample purification

Brush border membrane vesicles (BBMVs) were prepared from kidney and intestine using the Ca^2+^ precipitation method, and used for immunoblotting and Pi transport analyses, as described previously (Furutani et al., 2013; Ikuta et al., 2018; Schlingmann et al., 2016).

### 2.6 Transport assay

Transport of ^32^P into BBMVs was measured by the rapid filtration technique as described previously (Ikuta et al., 2018; Segawa et al., 2004). The transport rate of Pi into the kidney BBMVs was determined at 30, 60 and 120 s at 25°C with an inward gradient of 100 mM NaCl or 100 mM KCl and 0.1 mM KH_2_PO_4_ (pH 7.5). The Na^+^‐dependent Pi transport activity (activity rate in the presence of Na^+^ in the absence of Na^+^) is shown. All measurements were performed in triplicate.

Intestinal absorption was assessed on the basis of the ^32^P blood level after gavage of a test solution using a previously described protocol with modifications (Matsuo et al., 2005; Van Cromphaut et al., 2001). The test solution (pH7.4) contained 128 mM NaCl, 4.7 mM KCl, 2.5 mM CaCl_2_, 1.2 mM MgSO_4_, and 4 mM KH_2_PO_4_ (80 μCi/ml). For the study, 5 μl of the test solution per gram body weight was administered by gavage. Blood samples were obtained at the indicated time‐points and analyzed by liquid scintillation counting.

### 2.7 Immunoblotting

Protein samples were heated at 95°C for 5 min in sample buffer in the presence of 2‐mercaptoethanol and subjected to 8% sodium dodecyl sulfate‐polyacrylamide gel electrophoresis. The separated proteins were transferred by electrophoresis to Immobilon‐P polyvinylidene difluoride (Millipore) and treated with diluted antibodies. Signals were detected using Immobilon Western (Millipore).

### 2.8 Immunofluorescence staining

Mouse kidneys were fixed with the 4% paraformaldehyde solution (pH 7.2) overnight at 4°C, washed with phosphate buffered saline (PBS), cryoprotected with 10% and 20% sucrose at 4°C, embedded in OCT compound (Miles), and frozen in hexane at −80°C. Frozen sections (5 μm thick) were collected onto MAS‐coated slides (Matsunami Glass IND, Ltd.) and air‐dried (Ohkido, Segawa, Yanagida, Nakamura, & Miyamoto, 2003; Segawa et al., 2005). Serial sections were incubated with primary antibodies overnight at 4°C. Alexa Fluor 488 anti‐rabbit (Molecular Probes) and Alexa Fluor 568 anti‐mouse (Molecular Probes) were used as secondary antibodies for 60 min at room temperature. Thereafter, the sections were mounted with Dapi Fluoromount G^TM^ (Southern Biotech).

### 2.9 Antibodies

Rabbit anti‐Npt2a and Npt2c polyclonal antibodies were generated as described previously and used for immunoblotting and immunohistochemistry (Ohkido et al., 2003; Segawa et al., 2005). Mouse anti‐actin monoclonal antibody (Millipore) was used as an internal control. Horseradish peroxidase (HRP)‐conjugated anti‐rabbit or anti‐mouse IgG was utilized as the secondary antibody (Jackson Immuno Research Laboratories, Inc, West Grove, PA, USA). The diluted antibodies for immunoblotting were as follows: anti‐Npt2a (1:15,000), ‐Npt2c (1:1,500), and ‐actin (1:10,000). The diluted antibodies for immunofluorescence staining were as follows: anti‐Npt2a (1:500), ‐Npt2c (1:1,000), and ‐villin (Millipore) (1:500).

### 2.10 Histologic analysis

Mouse tissues were fixed with 4% paraformaldehyde overnight at 4°C and embedded in paraffin. Serial sections (5 µm thick) of several tissues were mounted on MAS‐coated slides (Matsunami Glass IND, Ltd.). The sections were treated for hematoxylin and von Kossa staining prior to light microscopic observations. The von Kossa staining for mineral deposits was performed by applying 5% silver nitrate to the sections and exposing them to bright light for 30 min (Schlingmann et al., 2016). The sections were slightly counterstained with hematoxylin.

### 2.11 Bone analysis

The femurs of all groups were fixed with 4% paraformaldehyde overnight at 4°C, decalcified for 4 weeks with 10% EDTA, and then, embedded into paraffin for immunohistochemical examinations. For von Kossa staining, tibiae were immersed in a mixture containing 2% paraformaldehyde and 2.5% glutaraldehyde diluted in a 0.067 M cacodylate buffer (pH 7.4) and post‐fixed with 1% osmium tetraoxide in a 0.067 M cacodylate buffer for 4 hr at 4°C. After post‐fixation, the tibiae were embedded in epoxy resin (Epon 812, Taab, Berkshire, UK) and sliced at a 500 nm thickness using by an ultramicrotome.

Immunohistochemical analyses of mouse bone sections were performed as described previously. Briefly, the sections were immersed into 0.3% H_2_O_2_ in methanol for 30 min to block endogenous peroxidase. To reduce nonspecific binding, 1% bovine serum albumin (BSA; Serologicals Proteins Inc.) in PBS (1% BSA‐PBS) was applied to the sections for 20 min. The sections were then incubated with rabbit polyclonal antisera against tissue‐nonspecific alkaline phosphatase (ALP) (Oda et al., 1999). We rapidly purified soluble forms of glycosylphosphatidylinositol‐anchored proteins using human tissue‐nonspecific ALP, or rabbit polyclonal anti‐dentin matrix protein‐1 (DMP‐1; Takara Bio) with 1% BSA‐PBS at room temperature for 2 hr as previously described, with some modifications (Oda et al., 1999). The thus treated sections were incubated with HRP‐conjugated anti‐rabbit antibody (Chemicon International, Temecula) for l hr, and thereafter, the immunoreactivities were visualized by using diaminobenzidine tetrahydrochloride as a chromogen.

For double detection of ALP and tartrate‐resistant acid phosphatase (TRAP), the sections immunostained for ALP were incubated with a mixture of 8 mg of naphthol AS‐BI phosphate (MilliporeSigma), 70 mg of red violet LB salt (MilliporeSigma), and 50 mM L(+) tartaric acid (0.76 g; Nacalai Tesque) diluted in 60 ml of a 0.1 M sodium acetate buffer (pH 5.0) for 20 min at room temperature. Methyl green was used to counterstain in all sections.

### 2.12 Statistical analysis

Data are expressed as means ± *SE*. Differences among multiple groups were analyzed by ANOVA. The significance of differences between the two experimental groups was established by ANOVA followed by Student's *t*‐test. A *p* value of less than .05 was considered significant.

## 3 RESULTS

### 3.1 Physiologic analysis, renal phosphate transport, and transporter expression in KLKO mice

**Figure 1:**
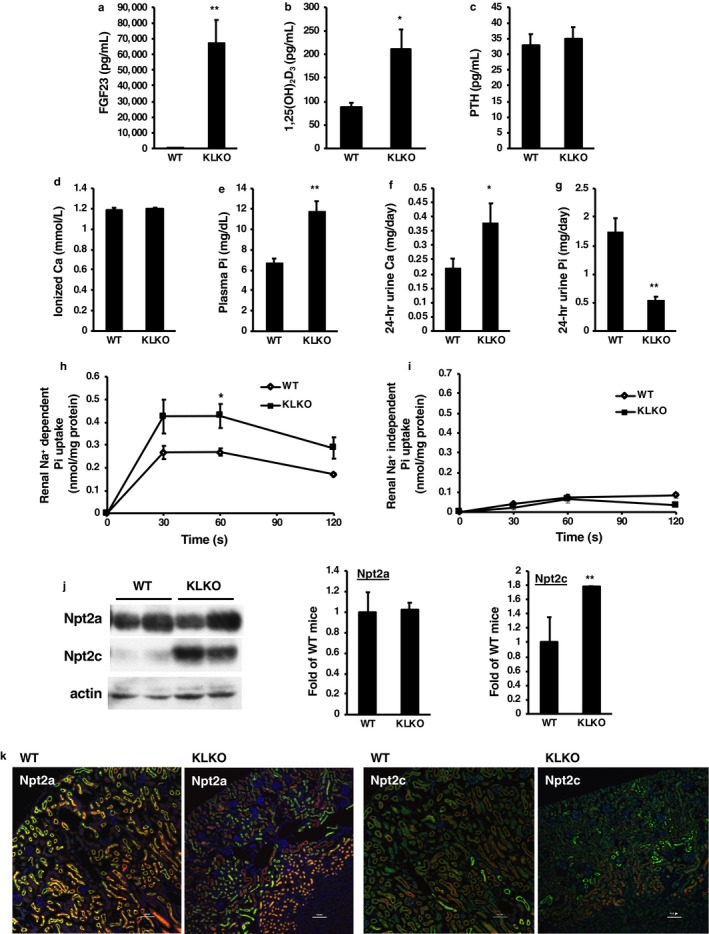
Physiologic analysis, renal phosphate transport, and transporter expression in KLKO mice. (a) Serum fibroblast growth factor (FGF)23, (b) plasma 1,25(OH)_2_D_3_, (c) plasma parathyroid hormone (PTH), (d) blood ionized Ca, (e) plasma Pi, (f) urinary Ca, and (g) urinary Pi excretion. Male mice at 8‐weeks of age (*n* = 5–20) were used. Values are mean ± *SE*. *p < .05, **p < .01. Metabolic cages were used for 24‐hr collection of urine from mice. (h) Renal Na^+^‐dependent and (i) ‐independent Pi transport activity in renal BBMVs isolated from the kidneys of 8‐week‐old WT and KLKO mice. Values are mean ± *SE*. *p < .05. (j) Western blot analysis of renal BBMVs isolated from the kidneys of 8‐week‐old WT and KLKO mice (*n* = 3–5). Each lane was loaded with 20 μg of BBMVs. Actin was used as an internal control. Relative intensity of Npt2a and Npt2c expression in WT mice was defined as 1.0. Values are mean relative intensity ± *SE*. **p < .01 versus WT mice. (k) Immunofluorescence staining of DAPI (blue), villin (red), and Npt2a or Npt2c (green) in kidney sections of 8‐week‐old WT and KLKO mice. Sections were prepared from kidneys embedded in OCT compound and frozen. Scale bar; 100 μm

The phenotypes of the KLKO mice were confirmed at 8 weeks of age (Figure 1). Compared with Klotho^+/+^ (WT) mice, KLKO mice exhibited high plasma concentrations of FGF23 and 1,25(OH)_2_D_3_, hyperphosphatemia, and hypercalciuria, but not ionized Ca or PTH, as described previously (Figure 1a–f) (Ohnishi et al., 2009a). Urinary Pi excretion was significantly lower in KLKO mice than in WT mice (Figure 1g).

Renal Na^+^‐dependent Pi transport activities were significantly higher in KLKO mice than in WT mice (Figure 1h). Na^+^‐independent Pi transport activities did not differ between WT and KLKO mice (Figure 1i). Npt2a protein expression levels did not differ significantly between WT and KLKO mice (Figure 1j). In contrast, Npt2c protein expression levels were significantly higher in KLKO mice than in WT mice (Figure 1j). Npt2a and Npt2c protein expression was confirmed by immunofluorescence staining (Figure 1k). Npt2a and Npt2c mRNA levels were not significantly different between WT and KLKO mice (Figure S1).

### 3.2 Biochemical analysis of KL2cDKO mice

Feeding of a low Pi diet can rescue the kl/kl mouse phenotype (Morishita et al., 2001; Segawa et al., 2007). In the present study, we confirmed the effects of a low Pi diet on KLKO mice (Figure S2). KLKO mice were fed a low Pi diet from 8 weeks of age. A low Pi diet increased the body weight and extended the lifespan of KLKO mice, similar to that of kl/kl mice (Figure S2a). After 8 days, a low Pi diet significantly decreased plasma Pi levels in KLKO mice compared with baseline levels before starting the test diet (Figure S2b).

**Figure 2:**
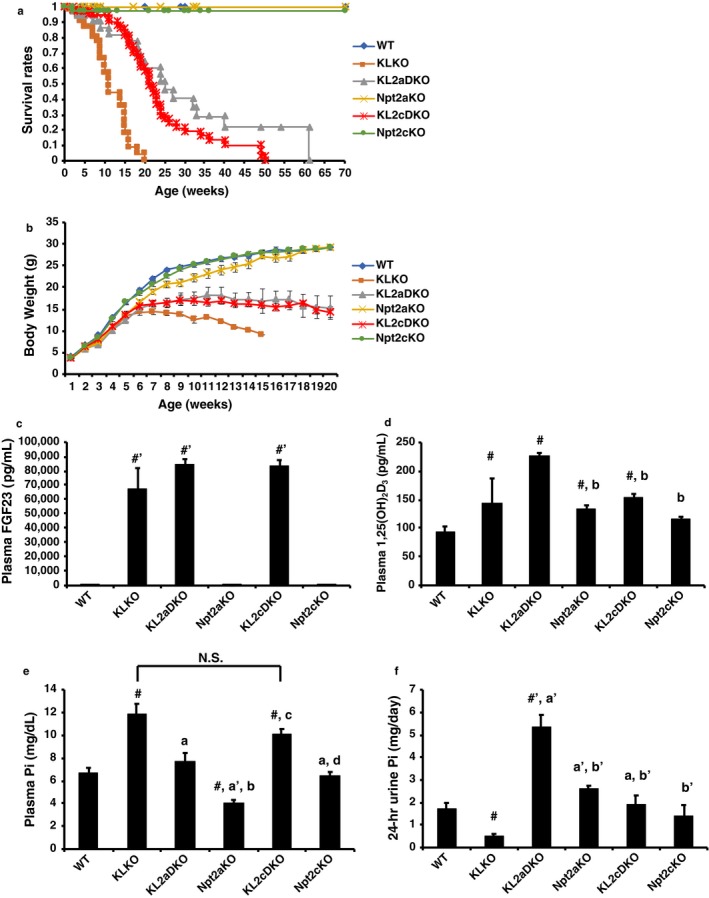
Characteristics of klotho/Npt2c double‐KO (KL2cDKO) mice at 8 weeks of age. 8‐week‐old male WT, KLKO, klotho/Npt2c double KO (KL2cDKO), and Npt2cKO mice. (a) Survival rates and (b) Growth curves for WT, KLKO, klotho/Npt2a double KO (KL2aDKO), Npt2aKO, KL2cDKO, and Npt2cKO mice. Body weight was measured every week. (c–f) (c) Plasma FGF23 (d) 1,25(OH)_2_D_3_, (e) Pi, and (f) urinary Pi excretion. Male mice at 8 weeks of age (*n* = 5–70) were used. Values are mean ± *SE*. ^#^ *p* < .05, ^#′^ *p* < .01 versus WT mice, ^a^ *p* < .05, ^a′^ *p* < .01 versus KLKO mice, ^b^ *p* < .05, ^b′^ *p* < .01 versus KL2aDKO mice, ^c^ *p* < .05 versus Npt2aKO mice, and ^d^ *p* < .05 versus KL2cDKO mice

To clarify the role of Npt2c in hyperphosphatemia in KLKO mice, we generated α‐klotho^−/−^Npt2c^−/−^ (KL2cDKO) mice for comparison with the α‐klotho^−/−^Npt2a^−/−^ (KL2aDKO) mice (Figure 2). Disruption of Npt2c in KLKO mice extended the lifespan of the KLKO mice (Figure 2a). KLKO mice had a significantly lower body weight than WT mice (*p* < .01 versus 4–14‐week‐old WT mice). KL2aDKO and KL2cDKO mice also had significantly lower body weights than WT mice (KL2aDKO; *p* < .01 versus 3–20‐week‐old WT mice, KL2cDKO; *p* < .05 versus 4–20‐week‐old WT mice). KL2aDKO and KL2cDKO mice had significantly greater body weights, however, than KLKO mice (KL2aDKO or KL2cDKO; *p* < .05 versus 12‐week‐old KLKO mice Figure 2b). The body weight gain was similar between KL2aDKO and KL2cDKO mice (Figure 2b).

Plasma FGF23, 1,25(OH)_2_D_3_, and Pi levels, and urinary Pi excretion levels of the mice at 8 weeks of age are shown in Figure 2c–f. The phenotypes of the KL2aDKO mice were consistent with the previous description (Ohnishi et al., 2009a). Plasma FGF23 levels were also high in KL2cDKO mice, similar to KLKO and KL2aDKO mice (Figure 2c). KLKO, KL2aDKO, Npt2aKO, and KL2cDKO mice had higher levels of 1,25(OH)_2_D_3_ than WT mice (Figure 2d). Real‐time PCR of renal 1αOHase and 24OHase mRNA was performed (Figure S3). The 1αOHase mRNA levels were significantly higher in KLKO, KL2aDKO, Npt2aKO, and KL2cDKO mice than in WT mice (Figure S3). In contrast, 24OHase mRNA levels were lower in KLKO, KL2aDKO, Npt2aKO, KL2cDKO, and Npt2cKO mice than in WT mice (Figure S3b). KL2aDKO mice had significantly lower plasma Pi levels than KLKO mice, as described previously (Figure 2e) (Ohnishi, Kato, Akiyoshi, Atfi, & Razzaque, 2011). The plasma Pi levels remained high in KL2cDKO mice, similar to that in KLKO mice (Figure 2f). Ohnishi et al. (2009a) did not measure urinary Pi excretion levels. In the present study, we measured urinary Pi excretion using metabolic cages. KL2aDKO mice had much higher urinary Pi excretion levels compared with the other groups of mice (Figure 2f). Urinary Pi excretion levels were significantly higher in KL2cDKO mice than in KLKO mice (Figure 2f). Hypercalcemia and hypercalciuria were also more severe in KL2aDKO mice compared with KLKO mice and the other mouse groups (Figure S3c and d). KL2cDKO mice did not exhibit hypercalcemia, but had higher levels of urinary Ca excretion compared with WT mice (Figure S3c and d). Urinary Ca excretion levels were not significantly different between KLKO and KL2cDKO mice (Figure S3c and d).

### 3.3 Disruption of Npt2c in KLKO mice recovered renal and vascular calcification

**Figure 3:**
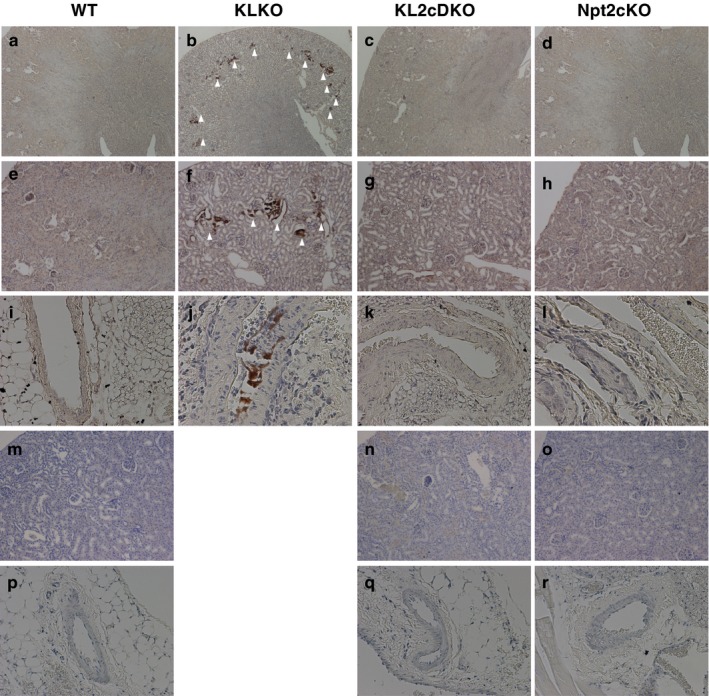
Disruption of ectopic calcification in KL2cDKO mice. The von Kossa staining of kidney (a–h, m–o) and aorta (i–l, p–r) sections from WT, KLKO, KL2cDKO, and Npt2cKO mice. Kidney and aorta sections were prepared from paraffin‐embedded tissues of 8‐week‐old (a–h, i–l) and 40‐week‐old mice (m–o, p–r), respectively. Counterstaining with hematoxylin is shown. White arrowheads indicate mineral deposits. Magnification; ×40 (a–d, m–o), ×200 (e–l, p–r)

Renal and vascular calcification was observed in KLKO mice at 8 weeks of age (Figure 3b, f and j), but not in WT (Figure 3a, e and i) and Npt2cKO (Figure 3d, h and l) mice on the basis of von Kossa staining, as described previously (Segawa, Onitsuka, Kuwahata, et al., 2009). Disruption of Npt2c in KLKO mice at 8 weeks of age abolished the renal and vascular calcification (Figure 3c, g and k). The kidneys and arteries of elderly mice were evaluated with von Kossa staining (Figure 3m–r). Plasma Pi levels of 40‐week‐old KL2cDKO mice also showed hyperphosphatemia (Plasma Pi concentration WT 7.36 ± 0.48 mg/dl, KL2cDKO 12.4 ± 0.75, Npt2c 7.49 ± 0.52, *n* = 4–8). No renal and vascular calcification, however, was observed in 40‐week‐old KL2cDKO mice (Figure 3n and q).

### 3.4 Npt2c plays an important role in KLKO mice during the juvenile period

**Figure 4:**
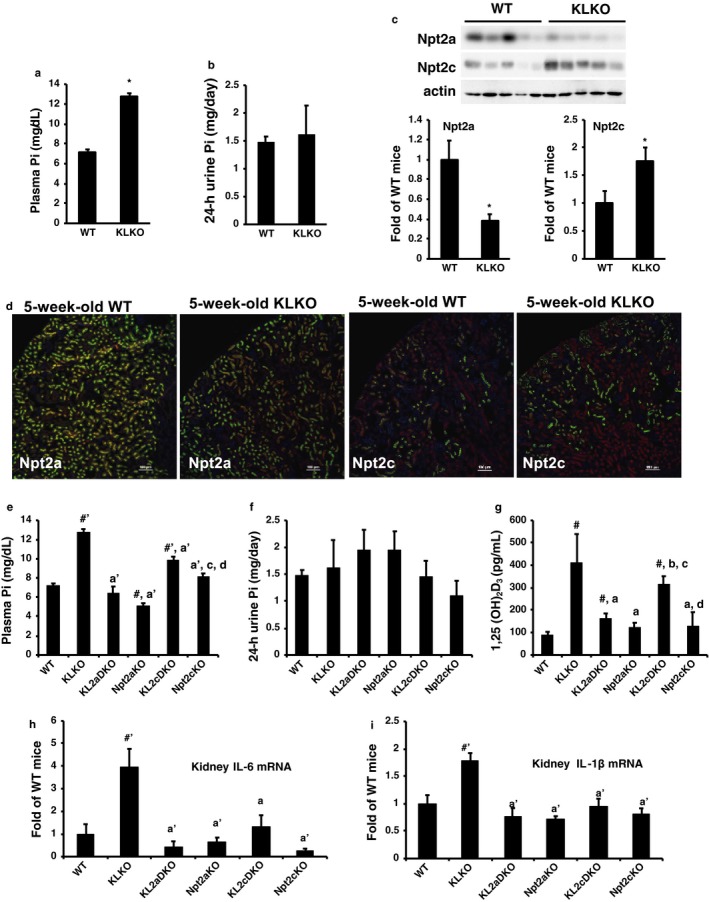
Characteristics of klotho/Npt2c double‐KO (KL2cDKO) mice at 5 weeks of age. (a) Plasma Pi and (b) urinary Pi excretion levels of 5‐week‐old WT and KLKO mice (*n* = 5–15). Values are mean ± *SE*. *p < .05. (c) Western blot analysis of renal BBMVs isolated from the kidneys of 5‐week‐old WT and KLKO mice (*n* = 5, respectively). Each lane was loaded with 20 μg of BBMVs. Actin was used as an internal control. Relative intensity of Npt2a and Npt2c expression in WT mice. Values are mean ± *SE*. *p < .05. (d) Immunofluorescence staining of Npt2a or Npt2c (green) in renal sections of 5‐week‐old WT and KLKO mice. DAPI (blue), villin (red). Sections were prepared from kidneys embedded in OCT compound and frozen. Scale bar; 100 μm. (e) Plasma Pi and (f) urinary Pi excretion, and (g) 1,25(OH)_2_D_3_ of 5‐week‐old mice (*n* = 5–9). Values are mean ± *SE*. ^#^ *p* < .05, ^#′^ *p* < .01 versus WT mice, ^a^ *p* < .05 versus, ^a'^ *p* < .01 versus KLKO mice, ^b^ *p* < .05 versus KL2aDKO mice, ^c^ *p* < .05 versus Npt2aKO mice, and ^d^ *p* < .05 versus KL2cDKO mice. (h) Interleukin (IL)‐6 and (i) IL‐1β mRNA levels in the kidney by real‐time PCR analysis. Male mice at 5 weeks of age (*n* = 5–9) were used. GAPDH was used as an internal control. Relative intensity of Npt2a and Npt2c expression in WT mice was defined as 1.0. Values are mean ± *SE*. ^#′^ *p* < .01 versus WT mice, ^a^ *p* < .05 versus, ^a'^ *p* < .01 versus KLKO mice

Because Npt2c is highly expressed during the juvenile period, we previously reported that Npt2c is a growth‐related Pi transporter (Ohkido et al., 2003; Segawa et al., 2002). KLKO mice at 5 weeks of age showed hyperphosphatemia and hypercalciuria, but not hyperphosphaturia or hypercalcemia (Figure 4a and b, and Figure S4a and b). The Npt2a protein expression levels were significantly lower in 5‐week‐old KLKO mice compared with WT mice (Figure 4c). In contrast, the Npt2c protein expression levels were significantly higher in 5‐week‐old KLKO mice compared with WT mice (Figure 4c). Furthermore, Npt2a and Npt2c protein expression in 5‐week‐old KLKO mice was confirmed by immunofluorescence staining (Figure 4d). Npt2a mRNA levels were significantly lower in KLKO mice than in WT mice at 5 weeks of age (Figure S4c). Npt2c mRNA levels, however, were not different between WT and KLKO mice at 5 weeks of age (Figure S4c).

Disruption of Npt2c in KLKO mice at 5 weeks of age significantly decreased the plasma Pi levels compared with KLKO mice, but plasma Pi levels were significantly higher in KL2cDKO mice than in WT and Npt2cKO mice (Figure 4e). Urinary Pi excretion did not differ significantly among groups (Figure 4f). Furthermore, KL2aDKO and Npt2aKO mice exhibited hypercalcemia and hypercalciuria compared with the other mouse groups (Figure S4d and e). KL2cDKO mice had slight, but significant hypercalcemia compared with WT and KLKO mice (Figure S4d). Furthermore, KL2cDKO mice had hypercalciuria compared with WT mice, but were not different from KLKO mice (Figure S4e). KL2aDKO and KL2cDKO mice at 5 weeks of age had higher 1,25(OH)_2_D_3_ levels than WT mice (Figure 4g). Furthermore, the 1,25(OH)2D3 levels were significantly suppressed in KL2aDKO mice, but not in KL2cDKO mice compared with KLKO mice (Figure 4g).

Real‐time PCR of renal 1αOHase and 24OHase mRNA was performed (Figure S4f and g). The 1αOHase mRNA levels tended to be higher in KLKO, KL2aDKO, Npt2aKO, and KL2cDKO mice than in WT mice (Figure S4f). In contrast, 24OHase mRNA levels tended to be lower in KLKO, KL2aDKO, Npt2aKO, KL2cDKO, and Npt2cKO mice than in WT mice (Figure S4g). Real‐time PCR on inflammation markers, interleukin (IL)‐6 and IL‐1β, are shown in Figure 4h and i. Disruption of Npt2c in KLKO mice significantly decreased the IL‐6 and IL‐1β mRNA levels to those in KLKO mice (Figure 4f and g).

### 3.5 Bone histochemical analysis

**Figure 5:**
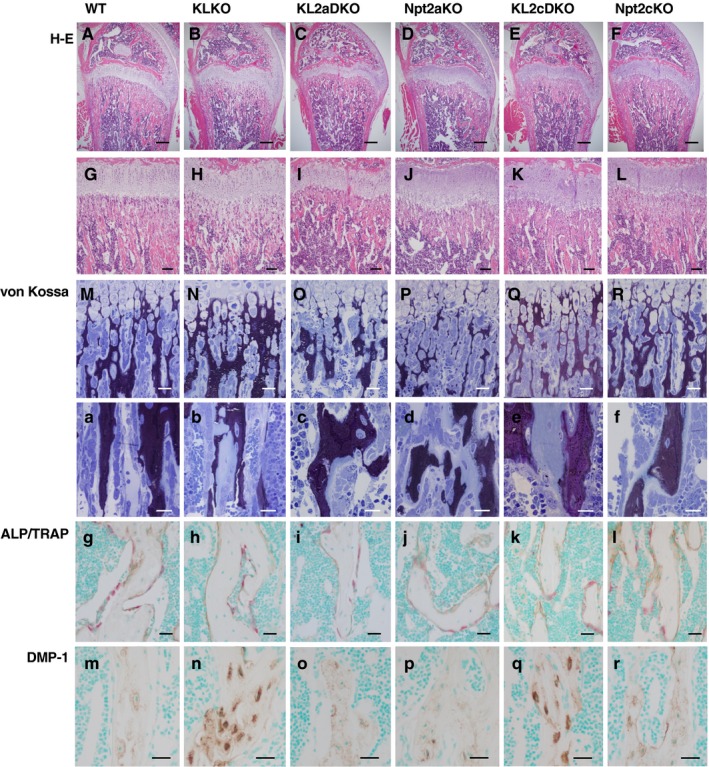
Bone analysis. Histologic analysis of longitudinal femoral sections from 5‐week‐old WT, KLKO, KL2aDKO, Npt2a, KL2cDKO, and Npt2cKO mice. (A–F) Hematoxylin/eosin (H‐E) staining of distal femurs in 5‐week‐old mice. Scale bar 300 μm. (G–L) Higher magnified images of distal metaphysis from H‐E staining. Scale bar; 100 μm. (M–R) Von Kossa staining (black) of metaphyseal trabeculae. Scale bar 30 μm. (a–f) Higher magnified images of von Kossa staining of metaphyseal trabeculae. Scale bar 20 μm. (g–l) Double staining of alkaline phosphatase (ALP, brown color) and tartrate‐resistant acid phosphatase (TRAP, red color) of metaphyseal trabeculae. Scale bar 30 μm. (m–r) Immunohistochemistry of DMP‐1 (brown color) in metaphyseal trabeculae. Scale bar 20 μm

Bone histochemical analysis was performed in 5‐week‐old mice (Figure 5). The bone volume of the femoral metaphyses in Npt2aKO and KL2aDKO mice seemed to be markedly reduced, while those in KLKO, Npt2cKO, and KL2cDKO mice appeared to be similar or slightly decreased compared with that in WT mice (Figure 5A–L).

Von Kossa staining to evaluate the metaphyseal mineralization in Npt2aKO and KL2aDKO mice revealed markedly reduced metaphyseal trabeculae in both groups (Figure 5O, P, c, d). Npt2aKO mice exhibited the broad areas of unmineralized bone matrix in the metaphyses, while KL2aDKO mice showed only slightly unmineralized bone matrix in the corresponding areas, indicating that bone mineralization of KL2aDKO mice seemed to have recovered to some extent by Npt2a deficiency (Figure 5O, P, c, d). Unlike Npt2aKO mice, mineralization in metaphyseal trabeculae was similar between Npt2cKO mice and WT mice (Figure 5M, R, a, f). Despite a similar metaphyseal bone volume as in WT, KLKO and KL2cDKO mice exhibited very large of unmineralized bone matrix compared with the WT mice (Figure 5M, N, Q, a, b, e).

Histologically assessment by double immunostaining for ALP/TRAP revealed similar numbers of ALP‐positive osteoblasts and TRAP‐reactive osteoclasts in all the groups (Figure 5g–l). Interestingly, an intense accumulation of DMP‐1 immunoreactivity was observed in the osteocyte lacunae of KLKO and KL2cDKO mice (Figure 5n and q). In contrast, Npt2aKO, KL2aDKO, and Npt2cKO mice showed a distribution pattern of DMP‐1 immunoreactivity in the osteocytic lacunar‐canalicular system that was similar to that of WT mice (Figure 5m, o, p, r).

### 3.6 Intestinal Pi absorption in KL2cDKO mice

**Figure 6:**
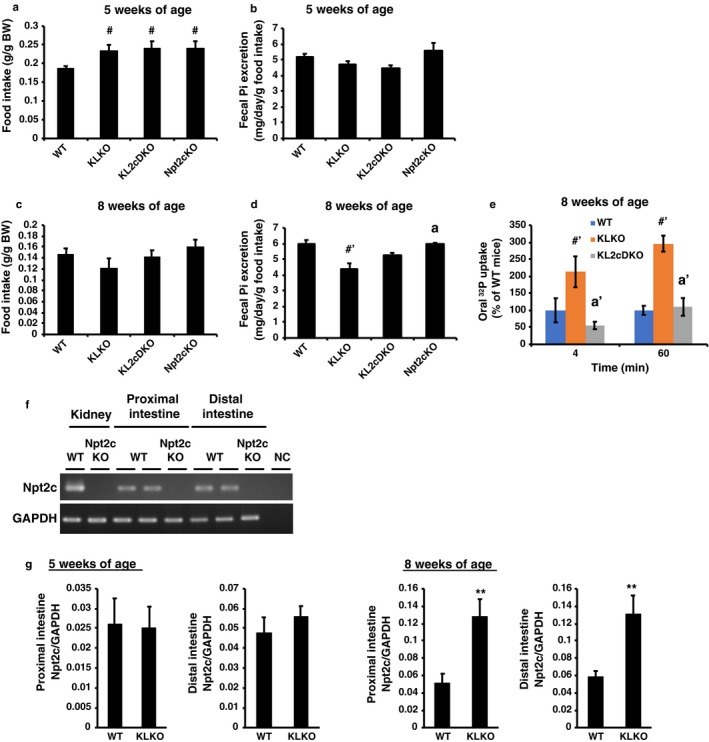
Disruption of Npt2c suppressed intestinal Pi absorption in KLKO mice. Food intake (a and c) and fecal Pi excretion (b and d). Metabolic cages were used for measurement of 24‐hr food intake (mg/g body weight [BW]), and collection of feces from mice (*n* = 5–9). Values are mean ± *SE*. ^#^ *p* < .05, ^#′^ *p* < .01 versus WT mice, ^a^ *p* < .05 versus KLKO mice. (e) Intestinal phosphate absorption assays in 8‐week‐old WT, KLKO, and KL2cDKO mice. Change in the blood Pi at 4 and 60 min after administration of the ^32^P test solution. Relative rate of ^32^P absorption in WT mice was defined as 100%. Values are mean ± *SE*, ^#′^ *p* < .01, ^a'^ *p* < .01, *n* = 3–4. (f) Npt2c mRNA in the kidney, proximal and distal intestine of WT mice. (g) Real‐time PCR analysis for Npt2c mRNA in the proximal and distal intestine of male WT and KLKO mice (*n* = 5–9). GAPDH was used as an internal control. Values are mean ± *SE*. **p < .01 versus WT mice

Intestinal Pi absorption in KLKO mice has not been studied in detail. KLKO mice may have increased intestinal Pi absorption due to the high plasma vitamin D levels. The high vitamin D levels may also contribute to hyperphosphatemia in KLKO mice. Fecal Pi excretion was measured in juvenile (5‐week‐old) and adult KLKO (8‐week‐old) mice using metabolic cages (Figure 6a–d). In the juvenile period, KLKO, KL2cDKO, and Npt2cKO mice had higher food intake (mg/g body weight) than WT mice, but there was no difference in fecal Pi excretion (Figure 6a and b). In adults, food intake (mg/g body weight) did not differ between groups, but fecal Pi excretion was significantly lower in KLKO mice compared with WT mice (Figure 6c and d). Disruption of Npt2c in 8‐week‐old KLKO mice, however, recovered the fecal Pi excretion level compared with 8‐week‐old KLKO mice (Figure 6d). Furthermore, to confirm the intestinal Pi absorption, ^32^P was administered orally and the Pi absorption rate was measured at 8 weeks of age (Figure 6e). The Pi absorption rates (^32^P transfer from intestine to the blood) were significantly increased in KLKO mice compared with WT mice (Figure 6e). Disruption of Npt2c in 8‐week‐old KLKO mice significantly decreased the Pi absorption rate compared with KLKO mice (Figure 6e).

Next, we investigated the reasons for the suppression of fecal Pi excretion in KL2cDKO mice compared with KLKO mice. Npt2c is mainly expressed in the kidney (Ohkido et al., 2003; Segawa et al., 2002). In the present study, we evaluated Npt2c in the mouse intestine. Npt2c mRNA expression was detected in the proximal and distal mouse intestine (Figure 6f). Furthermore, intestinal Npt2c mRNA expression levels were significantly increased in 8‐week‐old KLKO mice, but not in 5‐week‐old KLKO mice, compared with WT mice (Figure 6g).

**Figure 7:**
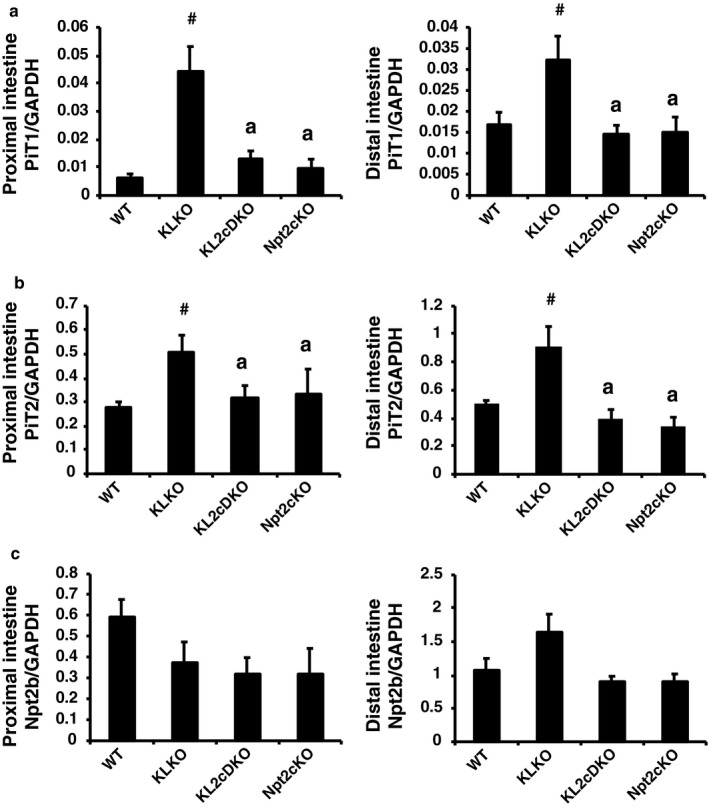
Intestinal Pi transporter in 8‐week‐old KL2cDKO mice. (a) PiT1, (b) PiT2, and (c) Npt2b mRNA levels in the proximal and distal intestine by real‐time PCR analysis. Male mice at 8 weeks of age (*n* = 5–9) were used. GAPDH was used as an internal control. Relative intensity of transporter expression in WT mice was defined as 1.0. Values are mean ± *SE*. ^#^ *p* < .05 versus WT mice, ^a^ *p* < .05 versus KLKO mice

Intestinal Pi transporter mRNA expression is shown in Figure 7. Intestinal PiT1 and PiT2 mRNA levels were significantly increased in KLKO mice compared with WT mice (Figure 7a and b). Intestinal Npt2b mRNA levels were not different in KLKO mice compared with WT mice (Figure 7c). Disruption of Npt2c in KLKO mice suppressed the induction of PiT1 and PiT2 mRNA (Figure 7a and b).

### 3.7 Role of Npt2c in controlling blood Pi levels in juvenile mice

**Figure 8:**
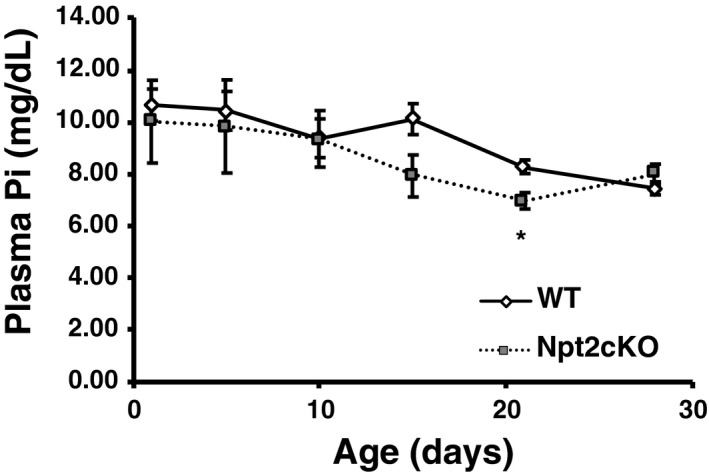
Npt2cKO mice had hypophosphatemia in the juvenile period. Plasma Pi concentration in WT and Npt2cKO mice at 1, 5, 10, 15, 21, and 28 days of age (*n* = 10–30). Values are mean ± *SE*, *p < .05 versus WT mice

Finally, we confirmed renal Npt2a and Npt2c protein expression around the juvenile period (Figure S5a and b). As described previously, Npt2a protein expression gradually increased with growth (Figure S5a and b). In contrast, Npt2c protein expression rapidly increased at 28 days of age (28D; Figure S5a and b). PTH and FGF23 levels were measured in mice at 15, 21, 28, and 60 days (Figure S5c and d). Plasma PTH levels in mice at 21, 28, and 60 days were significantly lower than those at 15 days (Figure S5c). In contrast, FGF23 levels in mice were lower at 21 and 28 days than at 15 and 60 days (Figure S5d). Next, we measured plasma Pi levels in juvenile Npt2cKO mice (Figure 8). In juvenile Npt2cKO mice, plasma Pi levels began to decrease around 15 days of age and showed significant hypophosphatemia at 21 days (Figure 8).

## 4 DISCUSSION

In the present study, to investigate the roles of Npt2c on FGF23/klotho signals in Pi homeostasis, we evaluated the KL2cDKO mouse phenotype. High plasma 1,25(OH)_2_D_3_ levels in KLKO mice may contribute to hyperphosphatemia by enhancing intestinal Pi absorption and renal Pi reabsorption. KLKO mice exhibited increased renal Npt2c protein levels. These phenotypes were also observed in kl/kl mice, but both Npt2a and Npt2c were increased in the kl/kl mice. The KLKO mouse phenotypes were similar to those described in several previous studies (Alexander et al., 2009; Hu et al., 2019, 2011; Kuro‐o et al., 1997; Morishita et al., 2001; Ohnishi et al., 2009a; Ohnishi, Nakatani, Lanske, & Razzaque, 2009b; Segawa et al., 2007; Tsujikawa et al., 2003; Yoshida et al., 2002).

The lifespan of both KL2aDKO and KL2cDKO mice was significantly extended compared with that of KLKO mice. In KL2aDKO mice, despite the increased plasma 1,25(OH)_2_D_3_ levels, we predicted that the increased renal Pi loss would reduce Pi toxicity and extend the lifespan, as well as improve soft‐tissue and vascular calcification compared with KLKO mice. In the present study, a low Pi‐diet prolonged the lifespan of KLKO mice. These observations are consistent with the reported phenotypes of KL2aDKO mice (Ohnishi et al., 2009a). In KL2aDKO mice, serum Pi levels are an important determinant of calcification and lowering serum Pi levels can reduce or eliminate soft‐tissue and vascular calcification, even in the presence of extremely high serum Ca and 1,25(OH)_2_D_3_ levels compared with KLKO mice.

Survival curves indicated that the lifespan of KL2cDKO mice was extended similarly to that of KL2aDKO mice compared with KLKO mice. The plasma Pi levels in adult KL2cDKO mice (8‐week‐old) did not decrease, however, and plasma 1,25(OH)_2_D_3_ levels also remained high. Renal Pi excretion was significantly increased in KL2cDKO mice compared with KLKO mice. In addition, adult (8‐week‐old) KLKO mice had significantly decreased fecal Pi excretion, and increased the levels of intestinal PiT1, PiT2, and Npt2c mRNA compared with WT mice. In vivo ^32^P oral administration, to KL2cDKO mice significantly suppressed intestinal Pi absorption compared with that in the KLKO mice. Thus, in KL2cDKO mice, although there was no change in the plasma Pi concentration, the body Pi load was reduced by increased intestinal and renal Pi excretion compared with that in KLKO mice. Such changes are expected to result in the suppression of calcification (kidney and aorta).

On the other hand, plasma Pi levels were reduced in 5‐week‐old KL2cDKO mice compared with KLKO mice. Renal Npt2c is specifically increased during the juvenile period, suggesting that Npt2c function is involved in renal Pi reabsorption. Urinary Pi excretion levels, however, were not altered in 5‐week‐old KL2cDKO mice compared with KLKO mice. Furthermore, previous and current data revealed no change in Pi excretion in 5‐week‐old Npt2cKO mice. We predicted that organs other than the kidney that express Npt2c contribute to the decreased plasma Pi concentration.

Deletion of Npt2c in KLKO mice did not have a clear recovery effect on bone phenotype. In addition, bone abnormalities in KLKO mice may have been caused by factors such as increased FGF23 due to klotho deficiency rather than suppression of plasma Pi levels, as described previously (Hikone et al., 2017).

Previous analyses of Npt2cKO mice (4 and 8 weeks of age) showed no clear abnormalities in Pi metabolism (Segawa, Onitsuka, Kuwahata, et al., 2009). Therefore, the role of Npt2c in the pathogenesis of HHRH due to Npt2c abnormalities is unclear in mice. We examined plasma Pi levels in Npt2cKO mice before weaning. Npt2cKO mice exhibited a significant decrease in plasma Pi levels at 21 days of age. We expect that Npt2c maintains postnatal plasma Pi levels. During the time when Npt2c is considered important, we evaluated the FGF23 and PTH concentrations, and found that FGF23 concentrations were significantly decreased. Changes in the plasma FGF23 levels may also play an important role in the induction of postnatal Npt2c protein.

The difference in the role of NaPi‐2c in humans and mice is not well understood. On the basis of our findings, mouse NaPi‐2c is necessary for increasing plasma Pi levels during the growth phase. In the mature period, it contributes to the absorption/excretion of Pi in the intestine/kidney rather than maintenance of the plasma Pi concentration, suggesting that it is involved in the retention of Pi in the body. On the other hand, human NaPi‐2c, like mouse NaPi‐2a, is expected to be an important molecule for maintaining plasma Pi levels. Although patients with HHRH typically present in childhood with rickets and/or nephrolithiasis, patients occasionally present as adults with low bone density (Dasgupta et al., 2014; Dhir, Li, Hakonarson, & Levine, 2017). NaPi‐2c may have different roles in the body depending on age.

**Figure 9:**
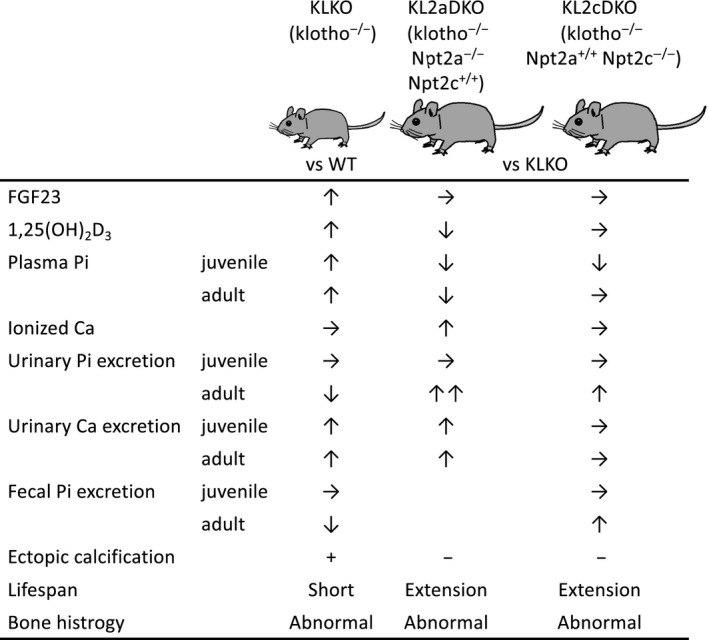
Phenotypes of KLKO, KL2aDKO, and KL2cDKO mice. Summary of the phenotypes of each mouse group inferred from the results are summarized in the Figure. We analyzed Pi and Ca metabolism in each mouse group using metabolic cages as shown in the Materials and Methods. Data are shown with respect to the increase or decrease in the value of each measurement compared with KLKO mice. Npt2cKO mice exhibited enhanced Ca excretion (Segawa, Onitsuka, Kuwahata, et al., 2009). No abnormalities in Ca excretion or plasma Ca concentration were observed in either KLKO mice or KL2cDKO mice. These results suggest that the phenotypic improvement in KL2cDKO mice compared with KLKO was not dependent on Ca or vitamin D metabolism, but on Pi metabolism

Finally, Npt2c may contribute to maintaining plasma Pi levels for growth during the juvenile period and may also be important for Pi retention in adults by controlling intestinal and renal Pi absorption. Figure 9 summarizes the KLKO, KL2aDKO, and KL2cDKO mouse phenotypes. Analysis of mouse NaPi‐2c may be useful toward understanding the role of NaPi‐2c in phenotypically different HHRH patients.

## CONFLICT OF INTEREST

None declared.
