# Supplementary material for: Role of sodium‐dependent Pi transporter/Npt2c on Pi homeostasis in klotho knockout mice different properties between juvenile and adult stages
Source: Physiol Rep. 2020 Feb 5;8(3):e14324. doi: 10.14814/phy2.14324 (PMC7002534; doi:10.14814/phy2.14324)
Supplement: Supplementary file 1 [file PHY2-8-e14324-s001.pptx]

## Slide 1
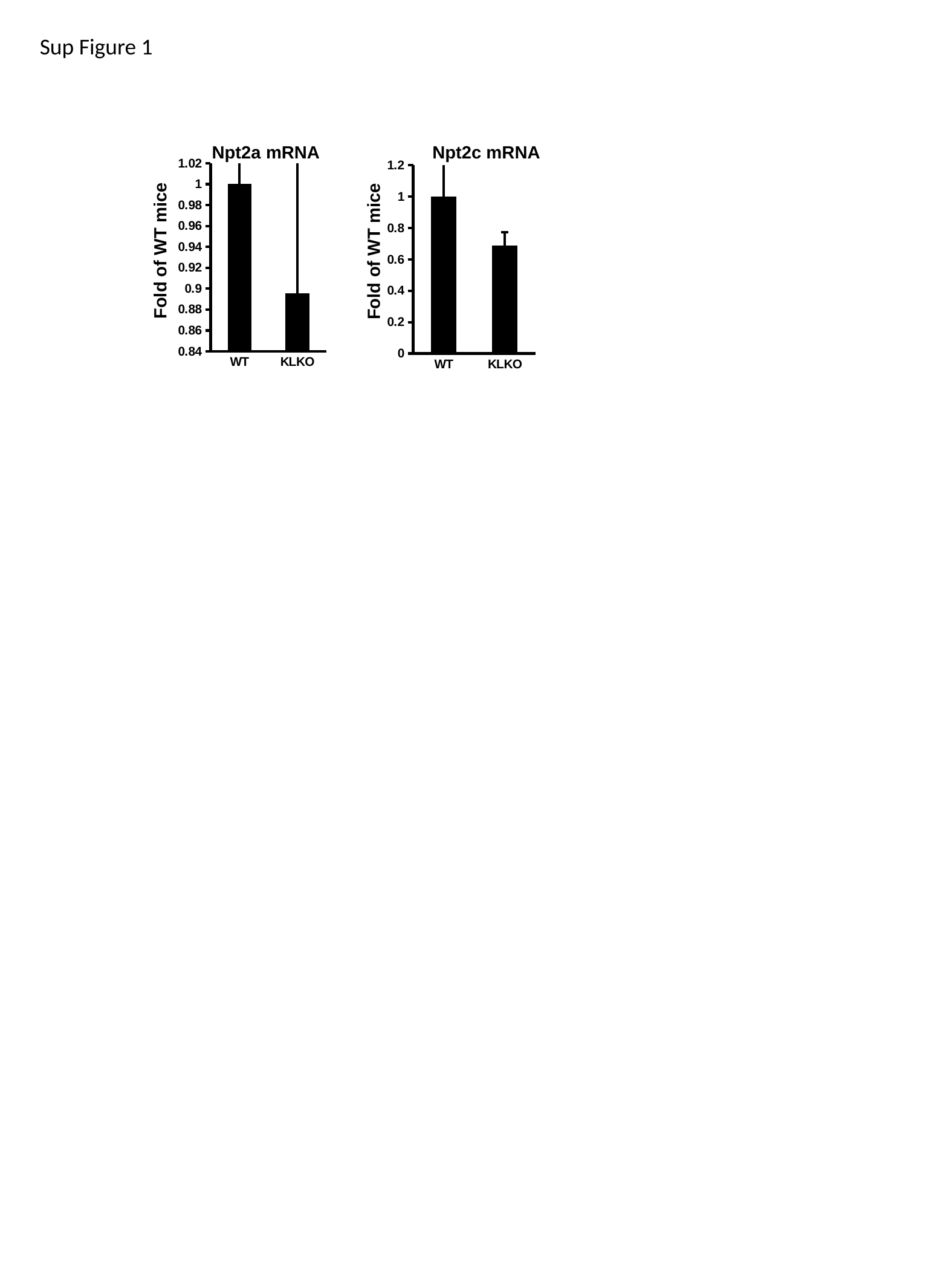

Sup Figure 1
Npt2a mRNA
Npt2c mRNA
### Chart
| Category | |
|---|---|
| WT | 1.0 |
| KLKO | 0.895439384515677 |
### Chart
| Category | |
|---|---|
| WT | 1.0 |
| KLKO | 0.685237425322662 |Fold of WT mice
Fold of WT mice

## Slide 2
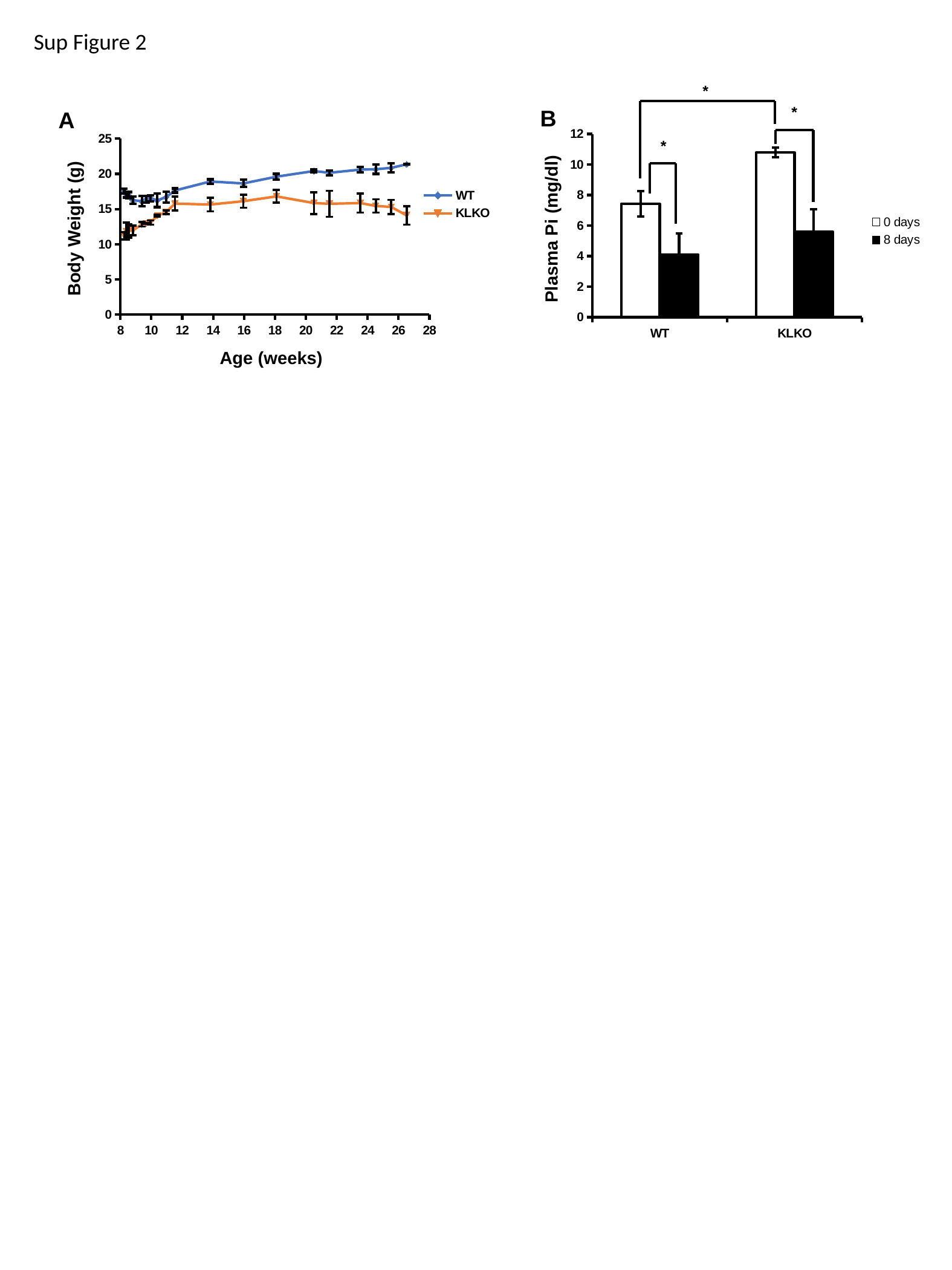

Sup Figure 2
*
*
B
A
### Chart
| Category | 0 days | 8 days |
|---|---|---|
| WT | 7.42825 | 4.101 |
| KLKO | 10.784666666666666 | 5.585333333333334 |
### Chart
| Category | WT | KLKO |
|---|---|---|*
Body Weight (g)
Plasma Pi (mg/dl)
Age (weeks)

## Slide 3
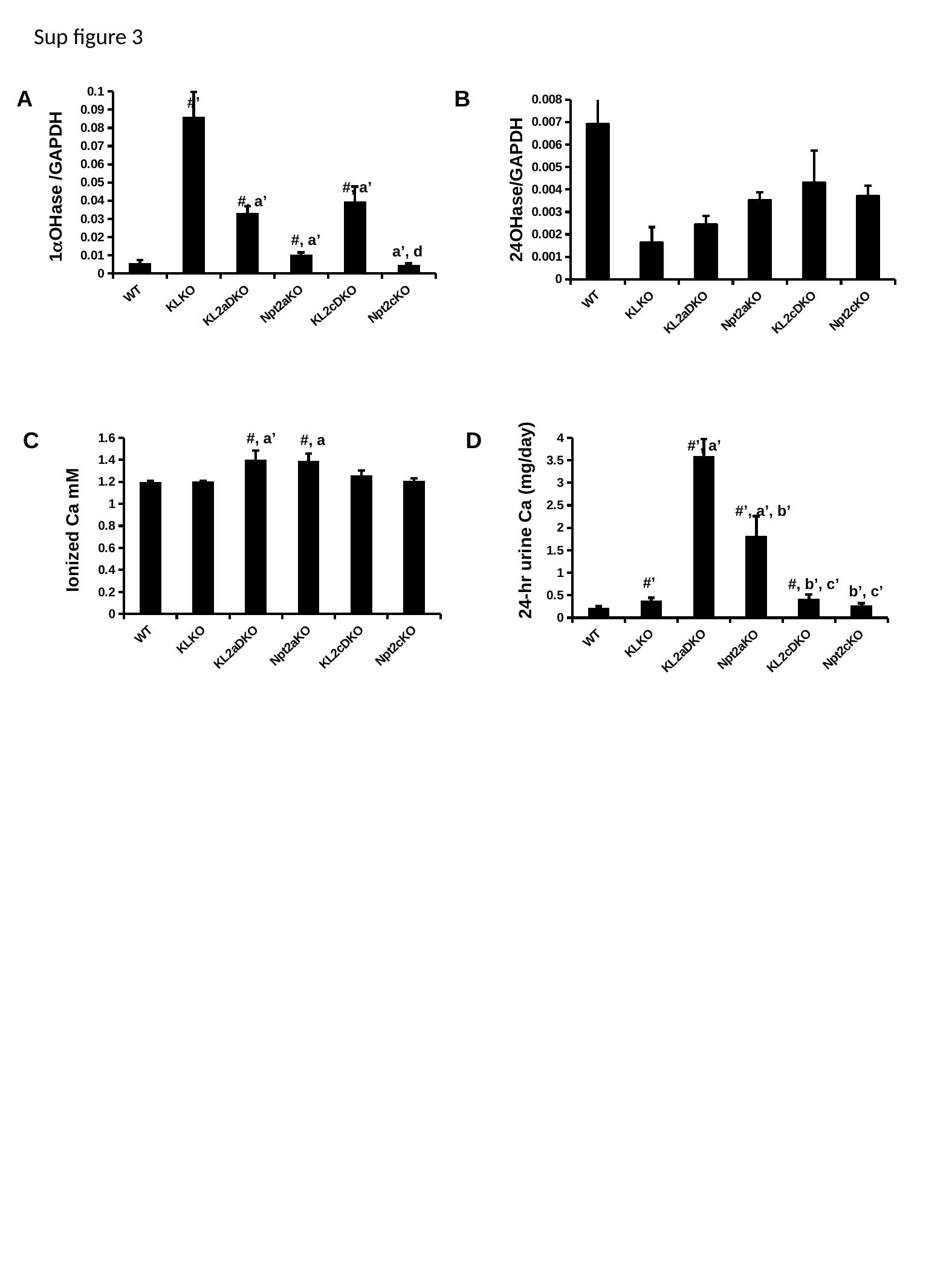

Sup figure 3
### Chart
| Category | |
|---|---|
| WT | 0.005588533617077022 |
| KLKO | 0.08601690460119964 |
| KL2aDKO | 0.03298143305117337 |
| Npt2aKO | 0.010360813989679031 |
| KL2cDKO | 0.039567163328703595 |
| Npt2cKO | 0.00442148820338801 |B
A
### Chart
| Category | |
|---|---|
| WT | 0.006921811679073773 |
| KLKO | 0.0016277355231600862 |
| KL2aDKO | 0.0024280685272585166 |
| Npt2aKO | 0.0035307744785273696 |
| KL2cDKO | 0.004298028304513229 |
| Npt2cKO | 0.0037200064403982765 |#’
1aOHase /GAPDH
#, a’
24OHase/GAPDH
#, a’
#, a’
a’, d
C
D
### Chart
| Category | |
|---|---|
| WT | 1.193240024293525 |
| KLKO | 1.20006075874104 |
| KL2aDKO | 1.399332048210018 |
| Npt2aKO | 1.387017382336116 |
| KL2cDKO | 1.257337755034945 |
| Npt2cKO | 1.205612246778623 |
### Chart
| Category | |
|---|---|
| WT | 0.2205525666666667 |
| KLKO | 0.37933294 |
| KL2aDKO | 3.5898672999999994 |
| Npt2aKO | 1.8216319199999997 |
| KL2cDKO | 0.42228748571428565 |
| Npt2cKO | 0.2642086 |#, a’
#, a
#’, a’
#’, a’, b’
24-hr urine Ca (mg/day)
Ionized Ca mM
#’
#, b’, c’
b’, c’

## Slide 4
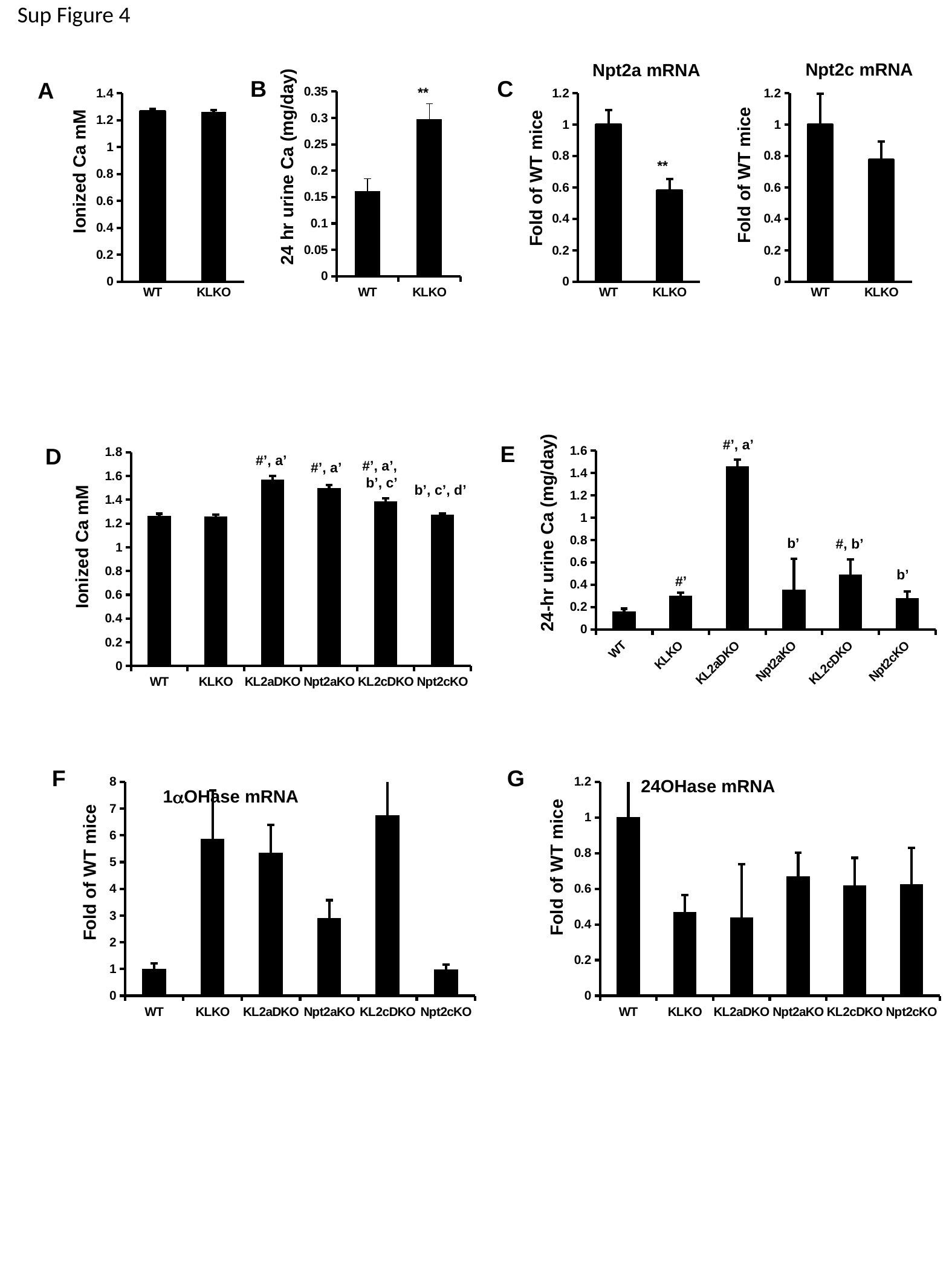

Sup Figure 4
Npt2c mRNA
Npt2a mRNA
B
C
A
**
### Chart
| Category | |
|---|---|
| WT | 0.1603748 |
| KLKO | 0.29710048 |
### Chart
| Category | |
|---|---|
| WT | 1.263833312241847 |
| KLKO | 1.2578497083379463 |
### Chart
| Category | |
|---|---|
| WT | 1.0000000000000002 |
| KLKO | 0.580115816894771 |
### Chart
| Category | |
|---|---|
| WT | 1.0 |
| KLKO | 0.7752867229780472 |24 hr urine Ca (mg/day)
**
Ionized Ca mM
Fold of WT mice
Fold of WT mice
#’, a’
E
D
### Chart
| Category | |
|---|---|
| WT | 0.1603748 |
| KLKO | 0.29710048 |
| KL2aDKO | 1.4603626 |
| Npt2aKO | 0.35277769999999997 |
| KL2cDKO | 0.48615273333333336 |
| Npt2cKO | 0.27937249999999997 |
### Chart
| Category | |
|---|---|
| WT | 1.263833312241847 |
| KLKO | 1.2578497083379463 |
| KL2aDKO | 1.5696782014383854 |
| Npt2aKO | 1.4945514427227993 |
| KL2cDKO | 1.385504058782033 |
| Npt2cKO | 1.270132623444645 |#’, a’
#’, a’,
b’, c’
#’, a’
b’, c’, d’
24-hr urine Ca (mg/day)
b’
#, b’
Ionized Ca mM
b’
#’
F
G
### Chart
| Category | |
|---|---|
| WT | 1.0 |
| KLKO | 5.85403782016507 |
| KL2aDKO | 5.343187214797144 |
| Npt2aKO | 2.8999213010597296 |
| KL2cDKO | 6.732432036982663 |
| Npt2cKO | 0.9827898463776542 |
### Chart
| Category | |
|---|---|
| WT | 1.0 |
| KLKO | 0.46948775985671654 |
| KL2aDKO | 0.43684756798249824 |
| Npt2aKO | 0.6673178260684882 |
| KL2cDKO | 0.6178959650357224 |
| Npt2cKO | 0.6261133949904665 |24OHase mRNA
1aOHase mRNA
Fold of WT mice
Fold of WT mice

## Slide 5
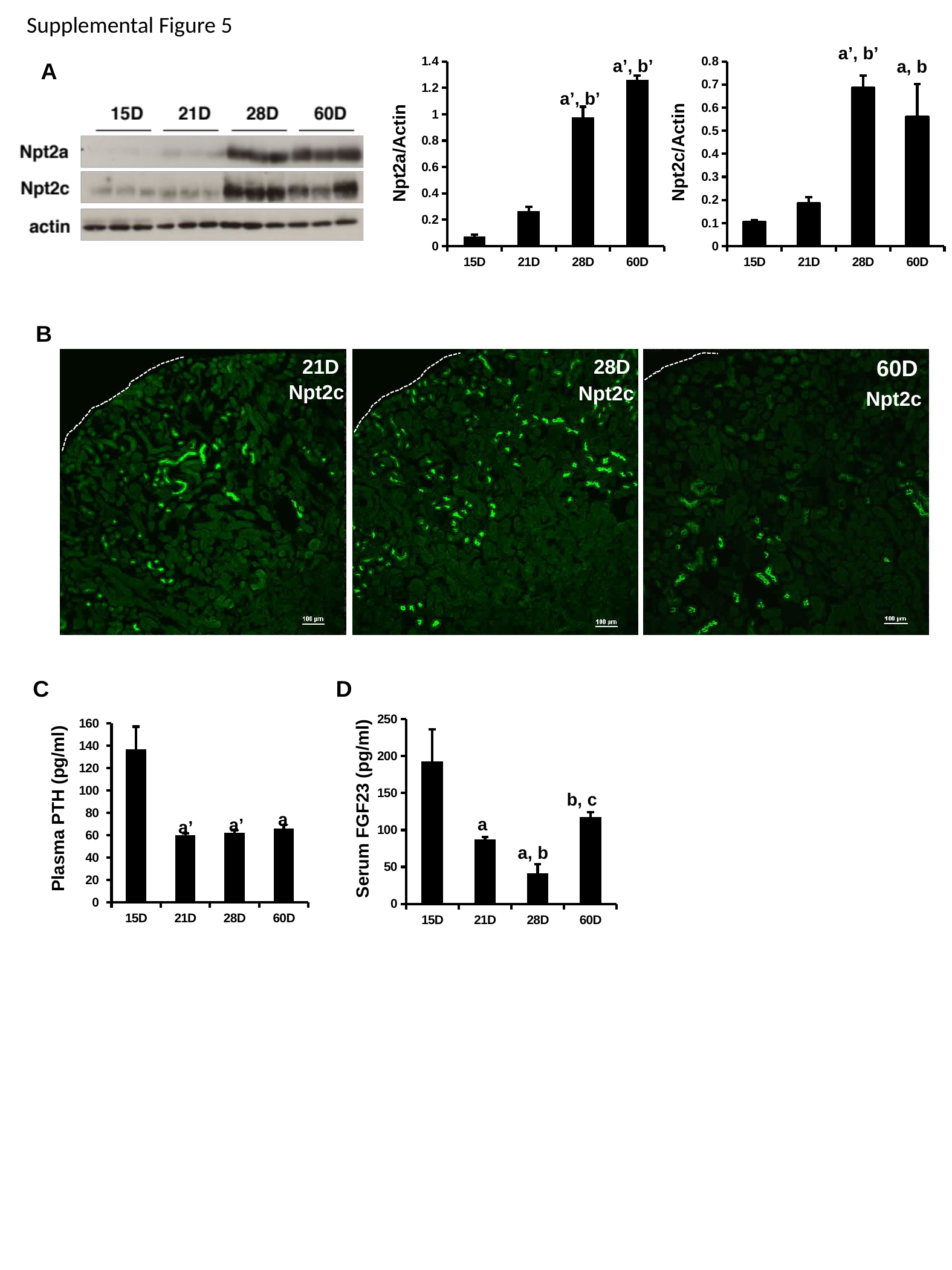

Supplemental Figure 5
a’, b’
a’, b’
### Chart
| Category | |
|---|---|
| 15D | 0.07028323226703674 |
| 21D | 0.2637025056326043 |
| 28D | 0.9740210021485485 |
| 60D | 1.2588979091840782 |
### Chart
| Category | |
|---|---|
| 15D | 0.10406927060827477 |
| 21D | 0.1855219057530443 |
| 28D | 0.6865580260828775 |
| 60D | 0.5599954938062363 |a, b
A
a’, b’
Npt2c/Actin
Npt2a/Actin
B
21D
28D
60D
Npt2c
Npt2c
Npt2c
C
D
### Chart
| Category | |
|---|---|
| 15D | 192.0726666666667 |
| 21D | 86.76700000000001 |
| 28D | 41.25 |
| 60D | 117.14333333333333 |
### Chart
| Category | |
|---|---|
| 15D | 136.58333333333334 |
| 21D | 59.875 |
| 28D | 62.16666666666667 |
| 60D | 65.6388888888889 |b, c
Plasma PTH (pg/ml)
Serum FGF23 (pg/ml)
a
a
a’
a’
a, b
